# Ontogenetic resource utilization and migration reconstruction with δ^13^C values of essential amino acids in the *Cynoscion acoupa* otolith

**DOI:** 10.1002/ece3.4471

**Published:** 2018-09-12

**Authors:** Kim Vane, Thomas Larsen, Barbara M. Scholz‐Böttcher, Bernd Kopke, Werner Ekau

**Affiliations:** ^1^ Leibniz Centre for Tropical Marine Research Bremen Germany; ^2^ Leibniz‐Laboratory for Radiometric Dating and Stable Isotope Research Christian‐Albrechts Universität zu Kiel Kiel Germany; ^3^ Institute for Chemistry and Biology of the Marine Environment University of Oldenburg Oldenburg Germany

**Keywords:** Amazon, carbon isotopes, life history, organic matrix, otolith chemistry

## Abstract

With the increasing anthropogenic impacts on fish habitats, it has become more important to understand which primary resources sustain fish populations. This resource utilization can differ between fish life stages, and individuals can migrate between habitats in search of resources. Such lifetime information is difficult to obtain due to the large spatial and temporal scales of fish behavior. The otolith organic matrix has the potential to indicate this resource utilization and migration with δ^13^C values of essential amino acids (EAAs), which are a direct indication of the primary producers. In a proof‐of‐concept study, we selected the Acoupa weakfish, *Cynoscion acoupa,* as a model fish species with distinct ontogenetic migration patterns. While it inhabits the Brazilian mangrove estuaries during juvenile stages, it moves to the coastal shelf as an adult. Thus, we expected that lifetime resource utilization and migration would be reflected in δ^13^C_EAA_ patterns and baseline values in *C. acoupa* otoliths. By analyzing the *C. acoupa* otolith edges across a size range of 12–119 cm, we found that baseline δ^13^C_EAA_ values increased with size, which indicated an estuarine to coastal shelf distribution. This trend is highly correlated with inorganic δ^13^C values. The δ^13^C_EAA_ patterns showed that estuarine algae rather than mangrove‐derived resources supported the juvenile *C. acoupa* populations. Around the juvenile size of 40 cm, resource utilization overlapped with those of adults and mean baseline δ^13^C_EAA_ values increased. This trend was confirmed by comparing otolith core and edges, although with some individuals potentially migrating over longer distances than others. Hence, δ^13^C_EAA_ patterns and baseline values in otoliths have great potential to reconstruct ontogenetic shifts in resource use and habitats. The insight could aid in predictions on how environmental changes affect fish populations by identifying the controlling factors at the base of the food web.

## 1 INTRODUCTION

Fish populations are increasingly influenced by human activities such as removal of estuarine vegetation for rural development, discharge of chemicals, climate change, and ocean acidification (Cheung, Brodeur, Okey, & Pauly, 2015; Halpern et al., 2008; Jackson, 2001; Nagelkerken, Russell, Gillanders, & Connell, 2015). To understand the impact of these habitat changes to the stability of fish populations, it is imperative to know which resources sustain them. By providing the resources at the bottom of the food web, primary producers are a major factor in determining the productivity at lower trophic levels and thus also predatory fish populations at higher trophic levels (Lynam et al., 2017; Pauly & Christensen, 1995). The use of resources can vary during fish life stages as is indicated by their utilization of distinct habitats or niches in an ecosystem (Huijbers, Nagelkerken, Debrot, & Jongejans, 2013; Kimirei et al., 2015). Moreover, fish actively undertake periodic migrations to synchronize their spawning time toward seasons with optimal resources for themselves and their offspring (Dingle & Drake, 2007; Nakazawa & Yamamura, 2006). Therefore, each life stage is sensitive to changes among primary producers, due to, for example, anthropogenic changes in the ecosystem, which affect the entire population. Shifts among primary producers affecting fish populations are not well characterized, as observational studies are often inconclusive in identifying key primary producers or habitat value for a fish species. Detecting a fish in a particular habitat does not directly identify the primary producers sustaining the food web it feeds on (Kruitwagen, Nagelkerken, Lugendo, Mgaya, & Bonga, 2010; Seitz, Wennhage, Bergstrom, Lipcius, & Ysebaert, 2014). This lack of knowledge also impairs effective conservation strategies for maintaining fish populations (Sheaves, Baker, Nagelkerken, & Connolly, 2015).

Characterizing resource utilization by juvenile stages is key for understanding population dynamics as they determine the recruitment of the adult population. How habitats are used by juveniles has been difficult to resolve and can be attributed to the complications of tracking especially small juveniles between habitats, which can occur periodically and over large spatial scales (Beck et al., 2001; Dahlgren et al., 2006; Gillanders, Able, Brown, Eggleston, & Sheridan, 2003). For this reason, fish otolith inorganic chemistry has been often utilized to qualify the movements of fish between habitats as otoliths provide a complete record of the fish's lifetime (Campana, 1999; Elsdon et al., 2008). The otolith calcium carbonate is accreted continuously from the early embryonic stage throughout the individual's life with often clear growth rings that are an indication of its age (Campana & Neilson, 1985). Simultaneously, the otolith calcium carbonate matrix is metabolically inert and retains the environmental chemistry in terms of minor and trace elements and their isotopes (e.g., strontium, barium, bulk δ^13^C, bulk δ^18^O). By interpreting elemental concentrations or isotopic values in the otolith, one could deduce whether the fish individual traversed distinct chemical environments such as freshwater and marine water habitats (Bath et al., 2000; Secor, Henderson‐Arzapalo, & Piccoli, 1995). However, there is growing evidence that the physiological and metabolic pathways of fish have implications for the elemental concentrations and bulk δ^13^C values in the otolith (Campana, 1999; Geffen, 2012; Radtke, Williams, & Hurley, 1987; Solomon et al., 2006; Sturrock et al., 2014). This causes uncertainty in the extent to which otolith inorganic chemistry can be interpreted for movement patterns and what is caused by physiological and metabolic changes (Chang & Geffen, 2013; Grønkjær, 2016).

The recent utilization of the otolith organic part, consisting primarily of proteins, could provide a more direct and unperturbed link between fish and its ambient environment (McMahon, Fogel, et al., 2011). Amino acids (AAs) are the building blocks of proteins, and about half of the 20 protein AAs are essential for metazoans, because they cannot synthesize them de novo (Howland et al., 2003). Therefore, metazoans depend on the essential amino acids (EAAs) through dietary sources. These EAAs can be traced to their biosynthetic origins such as algae, bacteria, fungi, and vascular plants. The carbon isotopic compositions or δ^13^C baseline values of EAAs, among primary producers, display geographical variations (i.e., isoscapes) and can therefore indicate fish movement and habitat connectivity (McMahon, Berumen, & Thorrold, 2012; Vokhshoori, Larsen, & McCarthy, 2014). Yet, each primary producer can have distinct biosynthetic pathways that lead to characteristic isotopic fractionations or δ^13^C patterns of individual EAAs (δ^13^C_EAA_) that can be used to identify the primary producer. These source diagnostic δ^13^C patterns or fingerprints of EAAs can be used as a marker of primary producers regardless of the δ^13^C baseline variations (Arthur, Kelez, Larsen, Choy, & Popp, 2014; Larsen et al., 2013). Thus, the otolith temporal increments and incorporated EAAs constitute an archive of lifetime resource utilization and migration through the individual fish life stages. However, only a limited amount of studies have looked into the potential of using the otolith organic matrix (Grønkjær et al., 2013; McMahon, Berumen, Mateo, Elsdon, & Thorrold, 2011; McMahon, Fogel, et al., 2011; McMahon et al., 2012).

The known distribution of the Acoupa weakfish, *Cynoscion acoupa*, from mangrove estuaries in the early life stages to offshore coastal shelf areas as adults (Barletta, Barletta‐Bergan, & Saint‐Paul, 1998; Barletta, Barletta‐Bergan, Saint‐Paul, & Hubold, 2003) suggests an ontogenetic migration between the two habitats and changes in resource utilization. Although it is a commercially important species occurring along the entire coast of Brazil, little is known about its foraging ecology or migration. Thus, δ^13^C_EAA_ baseline values and patterns from *C. acoupa* otoliths could provide more insight into its ontogenetic resource utilization and migration. We collected fish otoliths of *C. acoupa* individuals across a size range to identify habitat change and primary resource utilization along the ontogenetic development of this species. The main aim of this study was therefore to investigate the potential of δ^13^C_EAA_ analysis of otoliths to extract lifetime records of movement and resource utilization of *C. acoupa*. We tested whether baseline δ^13^C_EAA_ values in otolith edges of *C. acoupa* individuals at different lengths are consistent with the isotopic trend displayed by otolith inorganic δ^13^C values. We posit that δ^13^C_EAA_ patterns in combination with baseline δ^13^C_EAA_ values will reveal *C. acoupa* lifetime resource utilization and distribution from predominantly estuarine resources during juvenile stages to coastal shelf resources during mature stages. To test this, we analyzed otolith edges among different size classes and compared individual otolith cores and edges.

## 2 MATERIALS AND METHODS

### 2.1 Species and sampling location

The Acoupa weakfish, or *C. acoupa*, is a demersal fish that is caught by an artisanal fishery up to ~125 cm total length and weigh ~16 kg where adults are exclusively caught on the coastal shelf (Barletta et al., 1998; de Matos & Lucena, 2017). This sciaenid species occurs on the coastal shelf along the entire coast of Brazil and utilizes the mangrove estuaries as larvae and juveniles (Barletta et al., 2003; Barletta‐Bergan, Barletta, & Saint‐Paul, 2002a; Lima, Barletta, & Costa, 2015, Figure 1). In northern Brazil, *C. acoupa* spawns offshore at the onset and during the wet seasons and sexual maturation occurs ~40 cm (Almeida, Santos, Sousa, Carvalho Neta, & Andrade, 2016). It has a carnivorous diet, which consists mainly of shrimp and fish as well as small crustaceans and polychaetes in the juvenile stage (Ferreira et al., 2016).

Sampling of *C. acoupa* occurred only in Pará, northern Brazil, which receives a high annual precipitation of over 2,000 mm and leads to high freshwater discharges by the Amazon River. Consequently, in the wet season a high‐nutrient load is dispersed several kilometers offshore in a northward direction over a wide coastal shelf (Bustamante et al., 2015; Knoppers, Ekau, & Figueiredo, 1999; Smith & Demaster, 1996). The estuarine environment harbors extensive mangrove deltas and is strongly macrotidal with amplitudes of ~4 m (Schaeffer‐Novelli et al., 1990).

### 2.2 Sampling locations

Sampling of *C. acoupa* otoliths was conducted in Bragança, one of the main landing ports in the state of Pará, northern Brazil. Twenty juvenile *C. acoupa* individuals of 11–40 cm standard length (SL) were collected from local fishermen at the Caeté River, and 32 adults of 56–119 cm SL were sampled at the fish market in Bragança. Otoliths were removed, cleaned with demi water, and stored dry. Primary producers were collected at the Caeté River mouth close to where the juvenile fish were collected by the fishermen. No seagrass or coral reefs are found in this region. Leaves of the *Rhizophora mangle* mangrove tree, a rhodophyte growing on the mangrove roots *Bostrychia* sp., the freshwater algae *Spirogyra* sp., and degraded brown mangrove leaves, representing the bacterial community, were collected in the Caeté estuary. Coastal zooplankton, consisting predominantly of *Acartia tonsa*, was collected with a plankton net (mesh size 300 μm) to represent the coastal phytoplankton community. All primary producer samples were rinsed with MilliQ water, freeze‐dried, and stored in a −20° freezer.

### 2.3 Otolith preparation

Juvenile otoliths of *C. acoupa* individuals between 12 and 25 cm SL were used whole, cleaned in an ultrasonic bath with ultrapure water, and dried in the oven for 3 hr at 40°C. Subsequently, otoliths were homogenized with mortar and pestle and stored in a combusted glass vial. Otoliths of juvenile and adults between 25 and 119 cm were embedded in Araldite 2020 epoxy resin. Embedded otoliths were cut with a diamond saw through the center for a section of approximately 2 mm thickness. Sections were then glued with epoxy resin on a glass slide. Apart from an acetone wipe, the section surface was untreated to prevent contamination. The edges of these sectioned otoliths were micromilled for a 5 mg sample. To gain insight into resource utilization in the early life stage and latest life stage of seven individuals between 38 and 110 cm SL, the cores were micromilled alongside the otolith edges. Cores were micromilled according to the dimensions of the smallest otolith in the collection originating from a 12 cm SL *C. acoupa* individual.

### 2.4 Otolith bulk δ^13^C analysis

The inorganic calcium carbonate δ^13^C values from 42 homogenized *C. acoupa* otoliths and micromilled otolith edges were measured on a Finnigan MAT 251 gas isotope ratio mass spectrometer. This was connected to a Kiel III automated carbonate preparation device where samples react with phosphoric acid at 74°C. Data are reported in delta notation versus Vienna Pee Dee Belemnite standard (V‐PDB). The instrument was calibrated against the house standard (ground Solnhofen limestone), which in turn was calibrated against the NBS 19 standard reference material. Over the measurement period, the standard deviations of the house standard were 0.04‰ for δ^13^C values.

### 2.5 Otolith δ^13^C‐AA analysis

The 5 mg of all homogenized and micromilled otolith powder and 1 mg primary producer samples were hydrolyzed with 100 μl of 6M HCl per mg of sample in a microreaction vessel and 4 ml glass vial, respectively, with a reference of 20 μl 6‐aminocaproic acid at the same time. After flushing the vials with nitrogen, the samples were heated at 110°C for 20 hr. The acid was then evaporated in a heating block at 110°C for approximately 30 min. After acid hydrolysis, only the primary producer samples were purified with a DOWEX 50WX8 (hydrogen form, 100–200 mesh) column and eluted with 2M ammonium hydroxide. This was then evaporated to dryness in a 110°C aluminum block for approximately 1 hr. The samples were then redissolved in 200 μl of 0.1 M HCl, and 100 μl was transferred to a micro‐reaction vessel. All otolith and primary producer samples were then derivatized with the methoxycarbonyl esterification protocol (Walsh, He, & Yarnes, 2014). This included the addition of 35 μl methanol, 30 μl pyridine, and 15 μl methyl chloroformate. With a Hamilton syringe, 100 μl of chloroform was added, mixed, and rested for 5 min to separate into organic layers. The bottom chloroform layer was removed and transferred to a GC vial with 250 μl insert with a small amount of sodium sulfate. To prevent the sodium sulfate grains getting into the injector of the GC‐c‐IRMS, 40 μl was again transferred to a new GC vial with 250 μl insert and 4 μl of C_12:0_/C_20:0_ FAME standard was added.

A 2 μl aliquot of the sample was then injected in an Agilent 6890 GC with a VF‐WAXms column (30 m, 0.25 μm i.d., 0.25 μm film thickness) and interfaced to a MAT253 IRMS (Finnigan MAT) via a GC‐C II combustion interface. The GC temperature program utilized was 60°C (2 min), increased with 3°C/s to 245°C, and held for 20.3 min. All samples were injected via a cold injection system (CIS 4; Gerstel) in the splitless mode.

### 2.6 Data analysis

We obtained consistently good chromatography with sufficient chromatographic baseline separation across all samples for five EAAs, valine (Val), isoleucine (Iso), threonine (Thr), phenylalanine (Phe), and lysine (Lys). The measured δ^13^C_EAA_ values were corrected for kinetic fractionation during derivatization according to Docherty, Jones, and Evershed (2001). We followed a dual approach to present and evaluate the δ^13^C_EAA_ data; one based on baseline δ^13^C_EAA_ values (the corrected δ^13^C_EAA_ values) and the other on mean normalized δ^13^C_EAA_ values. The mean normalized values are calculated by subtracting each individual δ^13^C_EAA_ value from the mean δ^13^C values of all EAAs for each sample. While baseline δ^13^C_EAA_ values reflect spatial–temporal isotopic variation in the environment, normalized δ^13^C_EAA_ values track the biosynthetic origins of EAAs to algae, bacteria, fungi, and vascular plants independently of environmental conditions (Larsen et al. 2015). These source diagnostic δ^13^C_EAA_ patterns are also denoted as δ^13^C_EAA_ fingerprints as in Larsen, Taylor, Leigh, and O'Brien (2009); Larsen et al. (2013). To identify these EAA sources, we compared δ^13^C_EAA_ patterns between *C. acoupa* otoliths of individuals at different life stages and collected primary producers by performing multivariate analyses in R version 3.4.3 (R‐Development‐Core‐Team 2017). For assessing dietary contribution of primary producer sources to *C. acoupa* individuals, we applied principal component analysis (PCA; R: vegan) to the normalized δ^13^C_EAA_ values from otoliths and primary producers. We performed covariance matrix PCA to preserve variance as the range and scale of variables are in the same units of measure. We also created a classification model, linear discriminant function analysis (LDA, R: MASS), to determine the probability of dietary contributions to consumers. It is not necessary to mean normalize δ^13^C_EAA_ values prior to applying LDA, because the LDA procedure that scales observations to discriminant functions already normalizes them to ensure that within groups covariance matrix is spherical (Venables & Ripley, 2002). LDA is also useful for selecting the variables that are the most informative for discriminating between groups or classes. Similarly, univariate analysis performed on the output from multivariate analysis of variance (MANOVA, R: manova) can also be applied to test which dependent variables (δ^13^C_EAA_ values) are significantly different between groups (R: summary.aov). In conjunction with MANOVA, we used Pillai's trace to test the null hypothesis that groups have a common centroid in a dependent variable vector space. A rejection of this hypothesis entails that the groups have significantly different δ^13^C_EAA_ patterns or baseline values. To compare bulk inorganic δ^13^C values and mean baseline δ^13^C_EAA_ values, Spearman's correlation was applied due to non‐normally distributed data.

**Figure 1:**
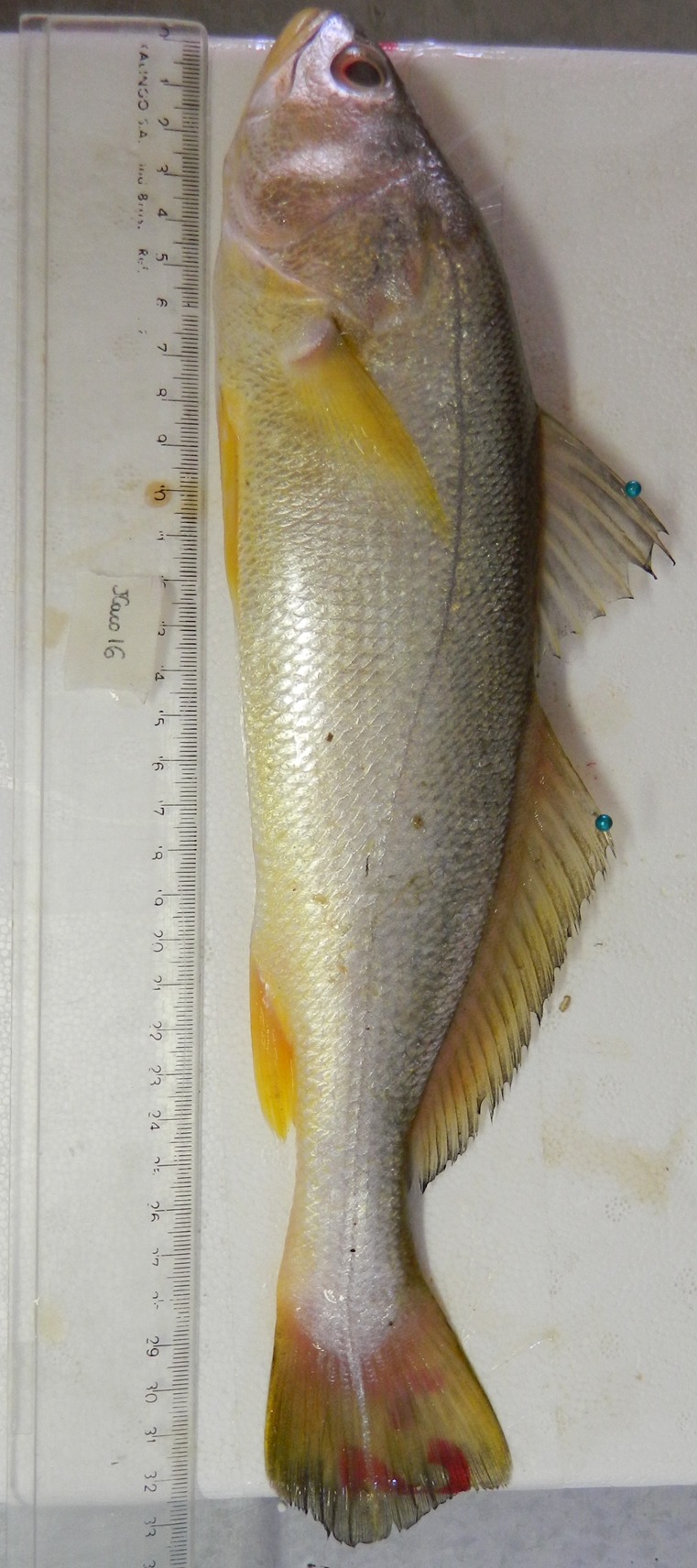
A juvenile *Cynoscion acoupa* of 32 cm total length from the Caeté River in Pará, Brazil

## 3 RESULTS

### 3.1 Otolith bulk δ^13^C versus mean δ^13^C_EAA_ values

**Figure 2:**
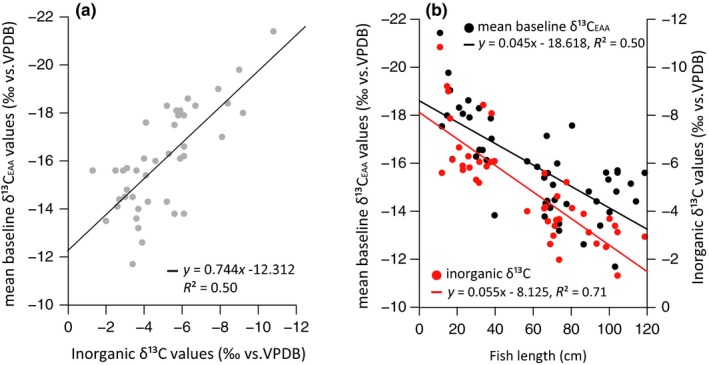
(a) Mean baseline δ^13^ C_EAA_ values versus the inorganic δ^13^C values measured in the same otolith material (*N* = 42). (b) Mean otolith baseline δ^13^ C_EAA_ values (*N* = 52) and a subsample of the same otoliths measured as inorganic δ^13^C values (*N* = 42) from individuals with a standard length from 12 to 119 cm

Inorganic δ^13^C values and mean baseline δ^13^C_EAA_ values in the same otoliths of *C. acoupa* individuals ranging between 12 and 119 cm SL showed the same trend of becoming more positive with increasing SL. These δ^13^C values were tightly correlated (*ρ* = 0.70, *p* < 0.001, Figure 2a), but with the inorganic fraction being 10‰ more enriched than the organic EAA fraction. Moreover, the range in mean baseline δ^13^C_EAA_ values was greater than that in bulk inorganic δ^13^C values (4‰ vs. 2.5‰ respectively, Figure 2b).

### 3.2 δ^13^C_EAA_ patterns in otoliths and primary producers

**Figure 3:**
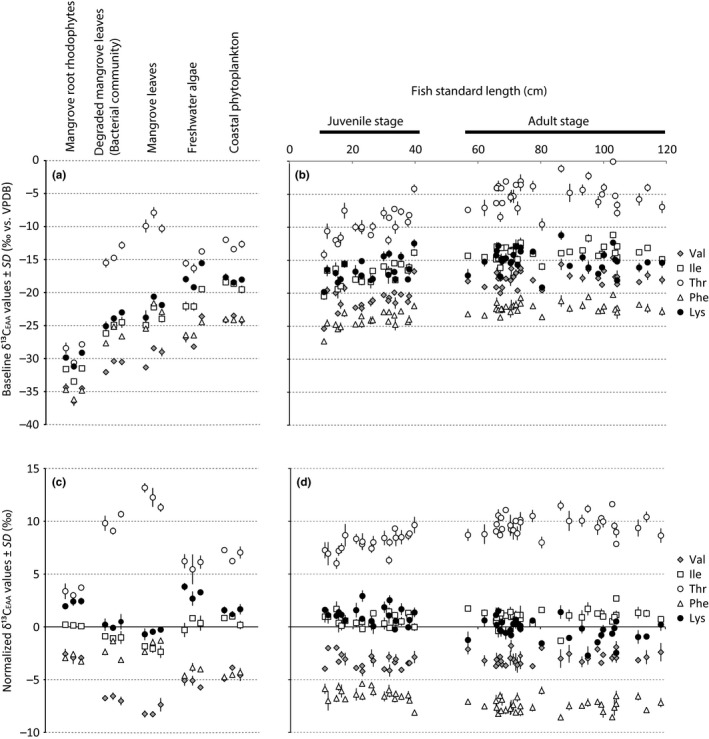
Individual baseline δ^13^C values of Val, Ile, Thr, Phe, and Lys with standard deviation from (a) primary producers and (b) whole fish otoliths from *Cynoscion acoupa* individuals with 12–25 cm SL and otolith edges from individuals with 25–119 cm SL. All measurements are based on triplicate injections. The mean normalized δ^13^ C_EAA_ values for (c) primary producers and (d) *C. acoupa* fish otoliths

Coastal primary producers as mangrove root rhodophytes and degraded and fresh mangrove leaves had overall more negative baseline δ^13^C_EAA_ values than the aquatic freshwater algae and phytoplankton (Figure 3a). Also, baseline δ^13^C_EAA_ values in otoliths from 12 to 119 cm SL individuals displayed an overall increasing trend from small to large individuals (Figure 3b) with juvenile (<40 cm) and adult *C. acoupa* groups (>55 cm) being significantly different (Pillai's trace = 0.67, *F*_5,47 _= 18.9; *p* < 0.001). For all EAAs, adult otolith edges were more ^13^C‐enriched than juvenile otoliths (*p* < 0.05).

**Figure 4:**
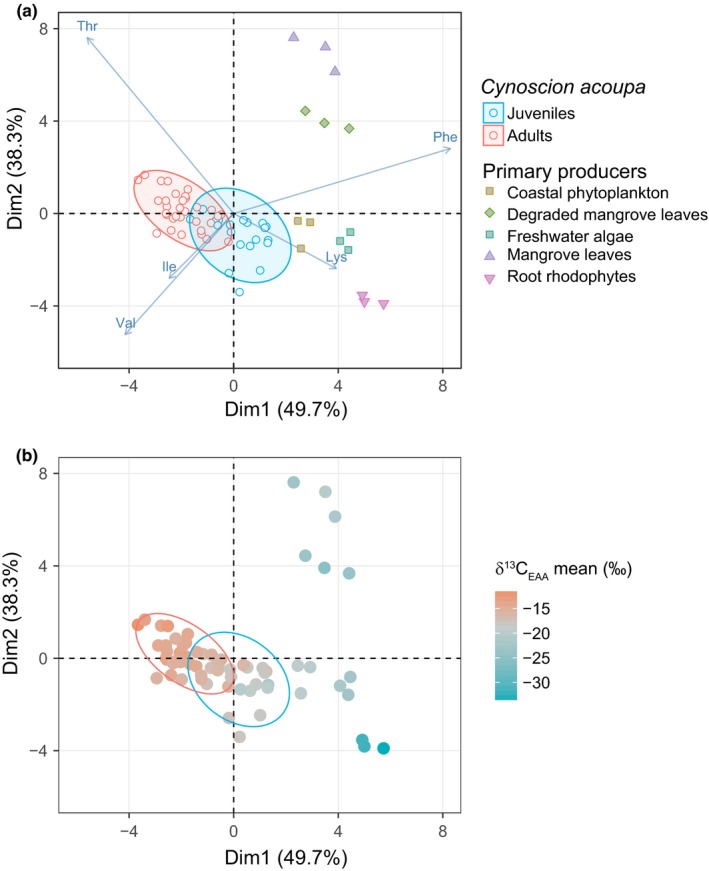
(a) Principal component analysis of δ^13^ C_EAA_ patterns in adult (*n* = 32) and juvenile (*n* = 20) otoliths (open symbols) and primary producers (filled symbols). Of the four principal components, the first two account for 88% of the variation (49.7% and 38.3%, respectively). (b) PCA coordinates of δ^13^ C_EAA_ patterns displayed simultaneously with baseline δ^13^ C_EAA_ values as a color gradient with warm colors signifying ^13^C‐enriched samples and vice versa for cool colors

We then investigated the extent in which baseline independent δ^13^C_EAA_ patterns can differentiate among otoliths of adults and juveniles and the various primary producer groups. The primary producers display particularly contrasting δ^13^C_EAA_ patterns in regard to Val, Phe, and Thr (Figure 3c). In comparison, δ^13^C_EAA_ patterns were less distinct between the juvenile and adult *C. acoupa* groups (Figure 3d). In the PCA (Figure 4a), Val, Phe, and Thr were strongly correlated with the first principal component separating freshwater from marine resources. To test whether juvenile and adult *C. acoupa* have different δ^13^C_EAA_ patterns, we followed a two‐step approach. We first identified and removed the EAAs that do not have significantly different mean normalized δ^13^C values between juvenile and adult otoliths, and then performed an MANOVA test with the remaining EAAs. After removing Ile and Val, the least informative variables for discriminating between adults and juveniles, we found that the juvenile and adult *C. acoupa* groups were significantly different (Pillai's trace = 0.55, *F*_3,49 _= 19.5; *p* < 0.001). According to the PCA (Figure 4a), Val and Ile were also the least correlated with the second principal component separating algal‐ and mangrove‐derived resources.

The PCA results showed that δ^13^C_EAA_ patterns of fish otoliths resembled coastal phytoplankton and freshwater algae more than mangrove root rhodophytes and degraded and fresh mangrove leaves. To visualize the distributional and resource utilization information that can be obtained from both mean baseline δ^13^C_EAA_ values and δ^13^C_EAA_ patterns, respectively, we constructed a PCA that incorporates both indicators. In the PCA based on δ^13^C_EAA_ patterns of adult and juvenile otoliths and the primary producers (Figure 4a), we color‐coded the principal component points according to their mean baseline δ^13^C_EAA_ values (Figure 4b). Red colors represent more positive baseline δ^13^C_EAA_ values and vice versa for blue colors (Figure 4b). The color gradient showed that adults have more positive baseline δ^13^C_EAA_ values than juveniles and coastal resources and that about half of the juveniles share baseline δ^13^C_EAA_ values with coastal resources (Figure 4b).

### 3.3 δ^13^C_EAA_ values in otolith core and edge

**Figure 5:**
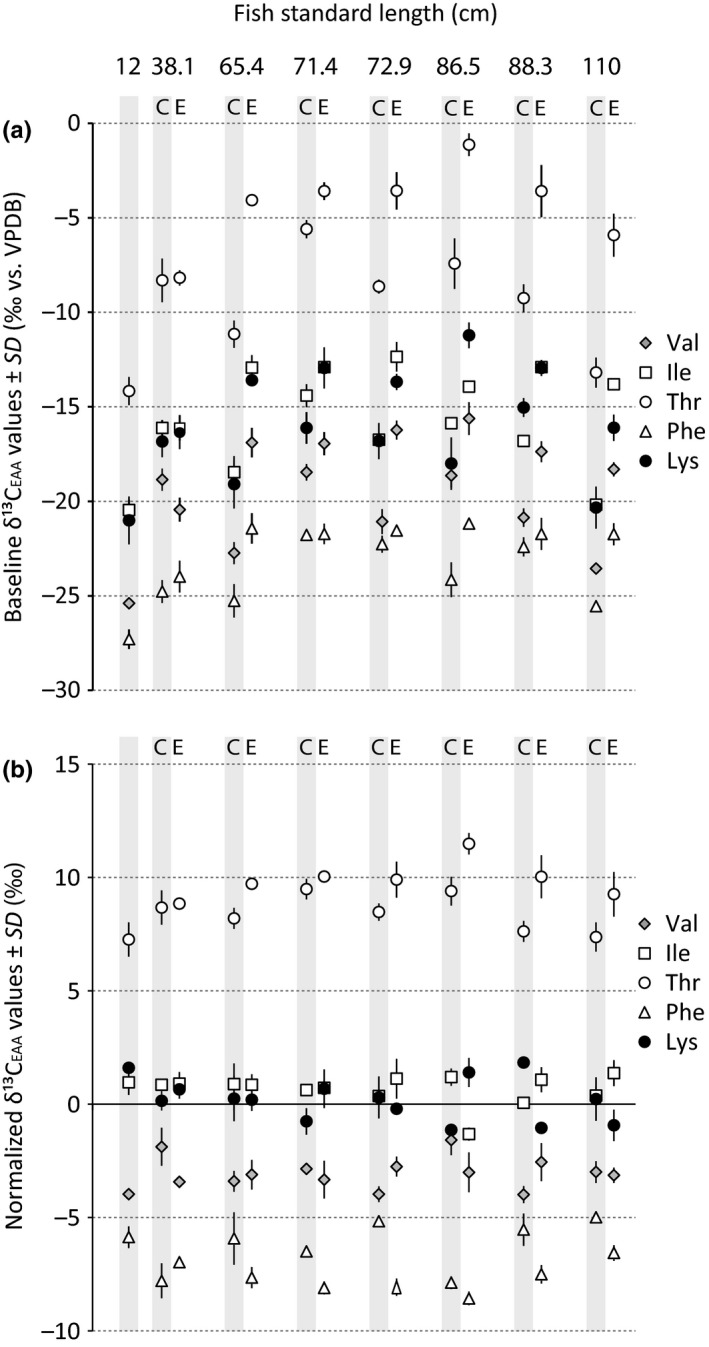
(a) A subsample of otoliths from differently sized individuals measured for baseline δ^13^ C_EAA_ values in core (C) and edge (E) of the otoliths. Otolith cores were microdrilled according to the dimensions of an otolith from an individual of 12 cm SL and are shaded in gray. (a) Individual δ^13^C values of Val, Ile, Thr, Phe, and Lys with standard deviation. (b) Calculated mean normalized δ^13^ C_EAA_ values from the otolith core and edges

The thick sectioning of northern *C. acoupa* otoliths did not allow us to detect incremental structures. Instead, we micromilled cores according to the otolith size dimensions of a 12 cm SL individual to get an insight into early and later life stages of seven individuals with 38–110 cm SL. Consistently lower baseline δ^13^C_EAA_ values were observed in the individual otolith cores than in the edges (ANOVA, *p* < 0.01 for Ile, Lys, Thr, Val; *p* < 0.05 for Phe; Figure 5a). We found that only Phe and Thr were significantly different between the two groups (ANOVA, *p* < 0.01; Figure 5b). This finding is consistent with Phe and Thr being among the most informative EAAs for distinguishing between juvenile and adult *C. acoupa* (Figure 4a). However, in a dependent variable vector space based on all five EAAs, it was indicated that core and edge otoliths had significantly different δ^13^C_EAA_ patterns (Pillai's trace = 0.97, *F*_5,8_ = 60.5; *p* < 0.001).

**Figure 6:**
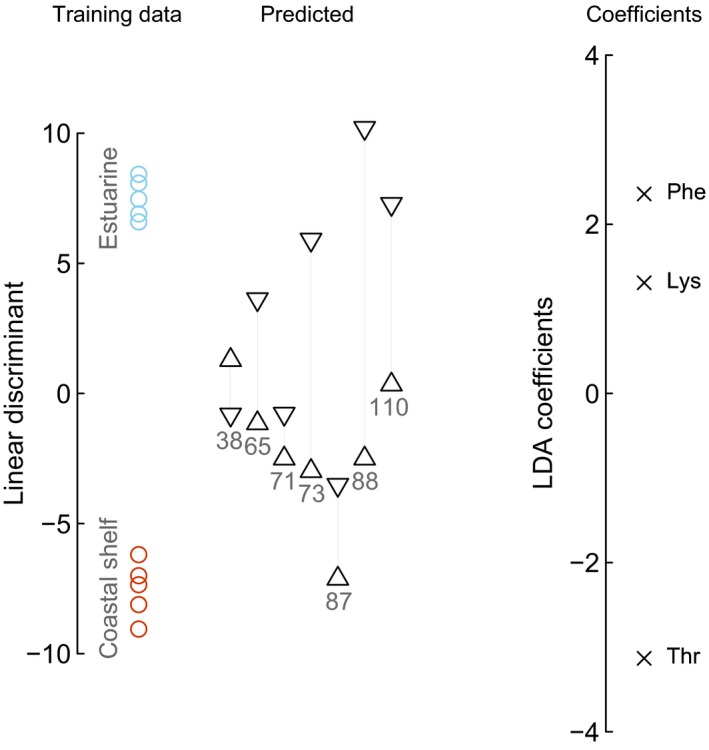
Linear discriminant function analysis for predicting use of coastal shelf and estuarine resources based on baseline δ^13^C values of Phe, Lys, and Thr, which are the independent variables displayed on the right as coefficients (crosses). Based on the training data comprising of juvenile and adult *Cynoscion acoupa* individuals, the model predicted that in most cases that EAAs from otolith cores (down‐pointing triangles) were more closely associated with estuarine resources than otolith edges (up‐pointing triangles), which were more associated with coastal shelf resources. Numbers under the triangles represent standard length in cm for each individual from which the otolith was obtained

To investigate individual lifetime use of estuarine and coastal shelf resources, we compared baseline δ^13^C_EAA_ values between core and edge otolith samples. For the training data, we selected five juveniles and five adults with the lowest and highest mean baseline δ^13^C_EAA_ values, respectively. As for the variables, we used the three most informative EAAs (Lys, Phe, Thr) for distinguishing between estuarine and coastal shelf resources. This classification model was applied to predict ontogenetic habitat shifts of seven *C*. *acoupa* individuals with different SL (Figure 6). Practically all individuals, excluding SL38, have core δ^13^C_EAA_ values that tended more toward estuarine resources than the edge measurements. However, some core or juvenile values, SL71 and SL87, overall inclined more toward coastal shelf baseline δ^13^C_EAA_ values and approach edge or adult values (Figure 6).

## 4 DISCUSSION

We obtained evidence of ontogenetic estuarine to coastal shelf migration by *C. acoupa* individuals from both inorganic δ^13^C and mean baseline δ^13^C_EAA_ values of otoliths. Yet, among individuals in juvenile and adult stages, considerable variations in both δ^13^C tracers were observed. Variations in δ^13^C value are not unexpected in tissues of a species residing in the highly variable Amazon region across a large distribution area with potential to move over considerable distances. In the wet season, the Amazon River disperses large amounts of freshwater into the coastal areas and several kilometers offshore toward the north (Loick‐Wilde et al., 2016; Weber et al., 2017). This can cause substantial spatial differences in δ^13^C baselines (Dittmar, Hertkorn, Kattner, & Lara, 2006) across areas which *C*. *acoupa* individuals traverse. These geographical, seasonal, and possibly long‐term or sudden changes in δ^13^C baseline values that can occur in the Amazon complicate the deduction of migration and resource utilization over long timescales as represented by otolith records. Disentangling metabolic change, diet, and DIC contributions to otolith inorganic carbon has been complicated (Elsdon, Ayvazian, McMahon, & Thorrold, 2010; Nelson, Hanson, Koenig, & Chanton, 2011; Solomon et al., 2006) and impeded the deductions about fish migration from otolith records (Jamieson, Schwarcz, & Brattey, 2004; Kalish, 1991; Schwarcz et al., 1998). Consequently, otolith inorganic δ^13^C trends are poor indicators for identifying δ^13^C baseline or resource changes. Source diagnostic δ^13^C_EAA_ patterns can overcome these δ^13^C baseline variations because δ^13^C_EAA_ patterns of primary producers remain largely constant despite the δ^13^C of the environmental conditions or spatial separation of primary producers (Larsen et al., 2009, 2013).

A pattern of lifetime resource utilization and distribution from estuary to coastal shelf by *C. acoupa* with increasing length was displayed by both δ^13^C_EAA_ baseline values and patterns. The PCA results depict that the resource utilization of juvenile and adult *C. acoupa* gradually become distinct and overlap at intermediate life stages, which could indicate that there is a regular movement between estuarine and coastal shelf environments by juveniles. An occurrence that is not unlikely in macrotidal Amazon estuaries where tides and body size of several fish species were observed to facilitate movement in and out of mangrove creeks (Brenner & Krumme, 2007; Krumme, Calderón, & Echterhoff, 2014). However, some otolith core measurements tended more toward coastal shelf resources and could imply that not all *C. acoupa* juvenile stages are spent within estuarine mangrove habitats. Estuarine resource use was also indicated by δ^13^C_EAA_ patterns in some otolith adult stages of *C. acoupa* although adults are not observed within estuaries. We speculate that some adults inhabiting the coastal shelf feed on fish that migrate out of the estuaries onto the coastal shelf. Visual census and a δ^13^C_EAA_ application also indicated that multiple species, which are believed to make ontogenetic migrations from mangrove or seagrass beds to coral reefs, also spend their early life stages in various habitats or complete their life cycle on offshore coral reefs (Kimirei et al., 2011; McMahon et al., 2012). Intrapopulation life history differences can be important for the population's persistence when facing environmental disturbances and can be a result of resource availability, food–predation risk trade‐offs, and spatial overlap of food webs (Bolnick et al. 2003; 2011).

Our results also show that coastal phytoplankton and freshwater algae contribute the most as resources to the juvenile *C. acoupa* community and that direct contributions from mangrove‐derived resources are negligible. This agrees with the spawning behavior of *C. acoupa* in North Brazil, which occurs with the rainy seasons (Almeida et al., 2016). Large numbers of larval and juvenile *C. acoupa* coincide with large blooms in zooplankton and shrimp populations in the mangrove estuaries, which often rely on planktonic and algal diets (Barletta‐Bergan, Barletta, & Saint‐Paul, 2002b; Barletta‐Bergan et al., 2002a; Ferreira et al., 2016; Lima et al., 2015). Phytoplankton blooms are also enhanced by the increased nutrient concentrations in the Amazon estuaries during the wet season (Dittmar & Lara, 2001; Santos et al., 2008; Smith & Demaster, 1996). However, other studies also indicated that mangrove carbon often does not directly contribute to sustaining the juvenile population (Igulu, Nagelkerken, van der Velde, & Mgaya, 2013; Kruitwagen et al., 2010; Melville & Connolly, 2003). Indirectly, mangroves can support fish populations by providing refuge to juvenile fish and supplying the coastal area with nutrients and mangrove carbon through the microbial and crustacean community (Bouillon et al., 2008; Dittmar et al., 2006; Nordhaus, Wolff, & Diele, 2006; Schories et al., 2003). Carbon from mangrove forests can therefore be an important, albeit indirect, factor in the primary production of coastal and offshore ecosystems supporting *C. acoupa* populations.

To gain a complete insight into the contribution of primary producers to estuarine and offshore fish food webs, all possible primary producers should be collected in the immediate environment and migration area of the species. This would also include the contribution of bacteria and fungi, which have been indicated to play an important role in the diet of first trophic‐level consumers (Pascal, Dubois, Goffette, & Lepoint, 2017; Steffan et al., 2017; Yi et al., 2017). Due to time and funding constraints, such samples were not included in the current study, but future studies could take full advantage of the source diagnostic ability of δ^13^C_EAA_ patterns to distinguish among algal‐, bacterial‐, fungal‐, and plant‐derived EAA sources (Larsen et al., 2013). While our results provide a general assessment of *C. acoupa* resource utilization, they concur with previous studies that also found insubstantial direct contributions of mangrove leaves to estuarine fish food webs (Igulu et al., 2013; Kruitwagen et al., 2010; Melville & Connolly, 2003) and the large direct contribution of aquatic algae such as phytoplankton and freshwater algae. This is a strong confirmation that δ^13^C_EAA_ patterns in otoliths are an indicator of lifetime resource utilization by individual fish.

In summary, δ^13^C_EAA_ measurements of the otoliths organic matrix display the resource utilization and migration of *C. acoupa* individuals. Baseline δ^13^C_EAA_ values indicated its known coast to offshore distribution pattern and source diagnostic δ^13^C_EAA_ patterns displayed a gradual change and overlap in resource utilization of *C. acoupa* juveniles and adults. The otolith organic matrix, hence, has great potential to be used as ontogenetic records with compound specific isotope analysis of δ^13^C in EAAs. As δ^13^C_EAA_ patterns of primary producers seem unaffected by δ^13^C baseline changes or environmental factors (Larsen et al. 2015), they could be utilized on archeological otoliths to identify changes in resource utilization at different life stages of past fish populations. A temporal comparison based on otolith archives could give us insight into how resource utilization by fish changes in response to habitat degradation and anthropogenic estuarine eutrophication.

## AUTHORS’ CONTRIBUTIONS

KV conceived the ideas and designed the methodology; KV, BSB, BK, and WE collected the data; KV and TL analyzed the data and led the writing of the manuscript. All authors contributed critically to the drafts and gave final approval for publication.

## DATA ACCESSIBILITY

Data available from the Dryad Digital Repository: https://doi.org/10.5061/dryad.8q7h4g7
